# Optimized Probability Sampling of Study Sites to Improve Generalizability in a Multisite Intervention Trial

**Published:** 2009-12-15

**Authors:** Carmen D. Samuel-Hodge, Jennifer L. Kraschnewski, Thomas C. Keyserling, Shrikant I. Bangdiwala, Ziya Gizlice, Beverly A. Garcia, Larry F. Johnston, Alison Gustafson, Lindsay Petrovic, Russell E. Glasgow

**Affiliations:** University of North Carolina at Chapel Hill; University of North Carolina at Chapel Hill, Chapel Hill, North Carolina; University of North Carolina at Chapel Hill, Chapel Hill, North Carolina; University of North Carolina at Chapel Hill, Chapel Hill, North Carolina; University of North Carolina at Chapel Hill, Chapel Hill, North Carolina; University of North Carolina at Chapel Hill, Chapel Hill, North Carolina; University of North Carolina at Chapel Hill, Chapel Hill, North Carolina; University of North Carolina at Chapel Hill, Chapel Hill, North Carolina; University of North Carolina at Chapel Hill, Chapel Hill, North Carolina; Kaiser Permanente Colorado, Denver, Colorado

## Abstract

**Introduction:**

Studies of type 2 translation, the adaption of evidence-based interventions to real-world settings, should include representative study sites and staff to improve external validity. Sites for such studies are, however, often selected by convenience sampling, which limits generalizability. We used an optimized probability sampling protocol to select an unbiased, representative sample of study sites to prepare for a randomized trial of a weight loss intervention.

**Methods:**

We invited North Carolina health departments within 200 miles of the research center to participate (N = 81). Of the 43 health departments that were eligible, 30 were interested in participating. To select a representative and feasible sample of 6 health departments that met inclusion criteria, we generated all combinations of 6 from the 30 health departments that were eligible and interested. From the subset of combinations that met inclusion criteria, we selected 1 at random.

**Results:**

Of 593,775 possible combinations of 6 counties, 15,177 (3%) met inclusion criteria. Sites in the selected subset were similar to all eligible sites in terms of health department characteristics and county demographics.

**Conclusion:**

Optimized probability sampling improved generalizability by ensuring an unbiased and representative sample of study sites.

## Introduction

Community-based research is vital for successful type 2 translation — adapting evidence-based interventions to real-world settings (1-4). However, study design and methods can limit the generalizability or external validity of many community-based randomized controlled trials, which often focus on the efficacy of the intervention (efficacy trials) (2,5,6). In contrast, practical clinical trials (PCTs) evaluate the applicability and generalizability of research by including representative participants, multiple and diverse settings, and a focus on measures relevant to decision makers (eg, cost, quality of life, participant reach, setting adoption) (7). PCTs can assess efficacious interventions for common conditions, such as obesity, because they provide information relevant to type 2 translation (5,6,8).

One essential element of a PCT is the use of diverse and representative settings and staff in the delivery of the intervention (9). Setting-level representativeness is as necessary for PCTs as patient-level representativeness, although it is ignored in most study reports (5,9). This feature of PCTs is often absent in community-based research because sites are frequently chosen by convenience sampling, on the basis of perceived site motivation or interest, proximity, or staff quality or resources, as opposed to probability sampling (8,10). In this way, convenience sampling can jeopardize conclusions regarding intervention effectiveness (11). Additionally, as opposed to regular clinic staff with competing demands and without special training, interventionists in non-PCTs are typically paid research staff, which further limits external validity (11).

We describe the process and outcomes of selecting sites for a research study designed to evaluate the type 2 translation of an intensive behavioral weight loss intervention designed for low-income women and conducted in county health departments. To improve study generalizability and meet PCT criteria, an optimized probability sampling protocol was used to select a representative sample of study sites for this project.

## Methods

### Study design

The study was divided into 2 phases: an assessment and preparation period (phase I) and a randomized controlled trial (phase II). The goals of phase I were to 1) identify, recruit, and select representative study sites; 2) evaluate stakeholder characteristics, resources, and experience relevant to weight loss interventions; 3) train staff at each of the sites to deliver the intervention; and 4) evaluate the process of preparing each of the participating sites. The primary aim of phase II, a randomized trial conducted at 6 county health departments with approximately 40 participants per site, was to assess the effectiveness of the intervention when implemented by health department staff in a community setting. This study reports on the first goal of phase I. Before site recruitment for phase I began, this component of the study was approved by the University of North Carolina institutional review board.

### Health department recruitment

The intervention in this study was designed for delivery by county health department staff, so our goal was to recruit a representative sample of health departments. North Carolina has 100 counties; most are served by county health departments (n = 79), and some are served by regional health districts (n = 21). For logistic reasons, participation was limited to counties whose health department was located within 200 miles of Chapel Hill and whose population was more than 10,000, which yielded 81 potential study sites (12).

Our recruitment efforts began with a presentation about the study at a meeting of North Carolina Public Health Incubator Collaboratives (http://nciph.sph.unc.edu/incubator/), which was attended by most county health directors. Application packets were distributed at this meeting (n = 17) or mailed to the health directors (n = 64) and included an informational brochure about the study, a memorandum of agreement, and an application form. We also mailed an invitation to the director of nursing at each potential site. Additionally, we circulated a program announcement through e-mail lists to health directors, nursing directors, health educators, and health departments that participate in the North Carolina Breast and Cervical Cancer Control Program and WISEWOMAN (Well-Integrated Screening and Evaluation for Women Across the Nation). Approximately 3 weeks after we distributed application packets, we contacted each health department via telephone to confirm receipt and answer questions.

Health departments were given approximately 6 weeks to complete the application form. We asked all 81 potential sites to respond to the application, even if they decided not to apply. For departments that did not return the packet, we attempted to follow up by telephone or e-mail at least 2 more times. Of the 81 potential sites, 13 did not respond, 25 indicated that they were not eligible to apply, 13 indicated that they were eligible but not interested, and 30 completed the application form and signed the memorandum of agreement (Table 1).

### Selecting study sites

Given the small number of sites (n = 6) that would make up the sample for the randomized trial, we felt that randomly selecting sites might not yield a representative sample of those eligible and interested or a logistically feasible sample (if many were located far from Chapel Hill). To ensure a representative and feasible study sample, we used an optimized probability sampling protocol to ensure the 6 health departments would have the following characteristics:

- No more than 1 health department from the same health district (21 counties are organized into large health districts that share staff; except in the case of health districts, health departments are organized at the county level, so these terms are used interchangeably).
- No more than 1 site with a bachelor's-level health educator (vs dietitian, registered nurse, or master's-level health educator) serving as the interventionist (only 4 of 30 counties had a bachelor's-level health educator, so we did not want to oversample this type of interventionist).
- At least 3 sites with at least a 30% racial/ethnic minority population (to ensure a reasonably large minority population in at least 50% of participating sites).
- Two sites from each tertile of county population (we wanted sites to be representative of small, medium, and large counties).
- No more than 1 health department located more than 150 miles from Chapel Hill (logistically, it would be difficult to conduct the study with several sites located more than 150 miles from Chapel Hill).

### Generating the probability sampling protocol

Using a SAS macro program (TS 498 Generating Combinations and Permutations, http://support.sas.com/techsup/technote/ts498.html), we generated all combinations of 6 counties from the 30 that agreed to participate (13,14). We then created a data set that listed only optimal combinations by including only the combinations that met all of the criteria outlined above. We used this set of combinations as the sampling frame and randomly chose 1 combination of counties by using SAS version 9.1.3 (SAS Institute, Inc, Cary, North Carolina), after specifying an initial seed for random number generation (14). If 1 of the selected health departments did not agree to participate or was not successful in enrolling the minimum number of participants, our plan was to identify the other optimal combinations that included the 5 participating health departments and select 1 combination at random from among them.

### Meeting with study sites

After the 6 study sites were selected, we scheduled an on-site meeting with the interventionist at each health department to provide an overview of the study, describe what participation would involve, and review compensation for participation. We also obtained their written consent to participate in a research study and asked that they complete 2 written surveys. The Health Department Capacity Survey is a 9-item written questionnaire administered to the health director or a designee. The survey asked questions about the health department's staffing and services, programs specific to adult weight management, and other resources. The Interventionist Survey asked about the interventionist's education and work experience, adult weight management experience, and perceived training needs. After this meeting, all 6 sites agreed to participate.

## Results

Health departments most commonly cited inadequate target population size as the reason that they were not eligible (Table 1). The most common reasons that health departments were not interested in participating were too many competing demands, self-assessed inadequate resources or capacity for program implementation, and self-assessed inadequate staffing.

From 30 eligible and interested sites, we calculated 593,775 possible combinations of samples of 6 sites (30!/[30 − 6]!/6!) (14,15). After applying the 7 criteria, 15,177 combinations were considered optimal and retained in the sampling frame, approximately 3% of the original possible combinations (Table 2). The most limiting criterion was having no more than 1 county 150 miles away. The least limiting criterion was requiring no more than 1 county in a health district.

Differences between departments by eligibility, interest, and selection for the study were generally small (Table 3). Not interested and not eligible sites were closer to Chapel Hill than were interested sites and nonresponders. Interested sites had larger populations on average than did the other groups. The mean percentage minority population was lower in nonresponders than in the other groups. However, the mean per capita income, percentage below poverty, and percentage enrolled in Medicaid varied minimally across groups. Nonresponding health departments were less likely to participate in the North Carolina Breast and Cervical Cancer Control Program or WISEWOMAN. These health departments also had smaller staffs on average and the smallest average county population.

The 6 selected sites' characteristics varied minimally from the 30 total sites that were eligible and interested (Table 3). The mean distance from Chapel Hill was shorter for selected sites than overall. The mean county population was also less, as was the mean number of health department staff. The staff positions were similar, with the exception that fewer of the selected sites had a registered dietitian. The mean percentage minority, per capita income, and percentage enrolled in Medicaid were similar between the groups.

Most of the selected sites (n = 5) offered patient education in diabetes, hypertension, and cholesterol in a group format. Additionally, most of the selected sites (n = 5) offered some type of adult weight management program, through either individual (n = 5) or group-based counseling (n = 4). Three sites reported collaborating or partnering with another agency to provide adult weight management services. Collaborating agencies included the Expanded Food and Nutrition Education Program (n = 5), faith-based organizations (n = 4), other state or local government agencies (n = 4), businesses (n = 3), employee groups (n = 3), hospitals or medical centers (n = 1), community health centers or clinics (n = 1), and YMCA/YWCA (n = 1).

All 6 interventionists had bachelor's degrees or higher. Half of the interventionists had substantial experience working in public health (Table 4). Similarly, the interventionists had been employed at their respective health departments for different periods: 3 were established (14-20 y), and 3 were new (1-3 y). Only 1 had received special training in adult weight management, although 4 had developed, implemented, or evaluated a weight management intervention. One-third had not been involved in a weight management program previously. Most had worked with the target population, low-income women aged 40 to 64 years, through health screening programs, minority health activities, or women's health promotion activities.

Interventionists were also asked to rate training topics. Topics that were rated most important included behavior change principles, weight management counseling, weight management program development, and community organization and mobilization. Least important topics included body mass index measurement and general physical activity and weight management recommendations and guidelines for adults. The most salient perceived barrier to implementing a weight management program at their respective sites was a lack of client interest (reported by 5 interventionists).

## Discussion

Using an optimized probability sampling method, we selected 6 study sites that were representative of the larger sample of 30 potential study sites. The SAS macro used to accomplish this has been described in the literature for obtaining balance in cluster randomized trials (13-15). One study that used this method was part of the Aid First Initiative in Baltimore, Maryland (14). This trial measured incidence rate of admission to treatment facilities for drug dependence after an intervention. Using the covariate-based constrained randomization allowed the investigators to obtain balance between census tracts (the unit of randomization) in terms of factors that could affect the outcome of interest, including geographic location, the percentage of vacant housing, and percentage of men employed (14). We have extended this approach to show that it is useful in selecting a probability sample of sites for participation in a type 2 translation clinical trial.

The major strength of using this technique is to improve external validity by increasing the representativeness of study sites and interventionists. This approach is distinctively different from convenience sampling (nonrandom site selection by the investigative team), which is most commonly used in multisite trials. Selection bias at the patient level is a risk that is often minimized in randomized controlled trials, but little research addresses this bias at the site level. The method described here addresses this bias by allowing for random selection from a set of eligible, interested sites.

An additional strength of the proposed approach is that it allows for the selection of a combination of sites that meet prespecified criteria (for example, distance from research center, percentage minority), ensuring study sites have desired characteristics and are logistically feasible. This method also allows an alternative site to be randomly selected if an initial site withdraws or is unable to enroll enough participants. Although this approach is similar to stratification, it allows more opportunity for similar sites to be chosen together by not forcing sites into strict strata. Stratification is also more difficult to implement when several factors define strata, especially when a small number of units is selected.

A major limitation in this study, and more generally in all clinical trials that focus on type 2 translation and enroll participants at multiple sites, is the lack of willingness of eligible and representative sites to participate. In this study, 43 of 81 potential counties were eligible, and of these, 30 (70%) were willing to participate. Because 30% of eligible sites did not agree to participate, our sample may not be fully representative of all potential study sites. An additional limitation is that only 3% of all combinations of 6 sites met our inclusion criteria. However, our approach ensures that from the identified 15,177 acceptable combinations of 6 study sites, an unbiased set was selected.

Enhanced external validity is key to type 2 translational studies and practical clinical trials (2,6,11). Translational studies should look not only at the representativeness of the participants but also at the participating settings and intervention staff. The optimized probability sampling method described here is useful in identifying an unbiased and representative sample of study sites.

## Figures and Tables

**Table 1:** Reasons Health Departments Were Not Eligible or Interested in Study Participation

**Reason^a^**	**No. of Health Departments**
**Not eligible**	25
Inadequate staffing: <1 full-time (or equivalent) permanent staff person working as a registered dietitian, health educator, or registered nurse assigned to patient education roles	7
Inadequate meeting space available: unable to accommodate a group of 20 women	5
**Inadequate target population size**	
<100 low-income women aged 40-64 y	13
<60% of low-income women aged 40-64 y English speaking	5
**Not interested**	13
Too many competing demands	8
Self-assessed inadequate staffing	5
Self-assessed inadequate resources or capacity for program implementation	7
Conflict with timing of program	4
Lack of interest by department staff	2
Too many barriers for eligible clients to participate in program	2
Already have a weight management program in place	1

a Health departments could select >1 reason.

**Table 2:** Number and Percentage of Site Combinations Meeting Individual and Combined Criteria of 593,775 Possible Combinations

**Criterion**	No. (%) of Combinations That Met Criteria
≤1 County 150 miles away	121,380 (20)
≤1 County in a district	569,250 (96)
≤1 County with a staff member with a bachelor's degree as health educator/nutritionist	493,350 (83)
≥3 Counties with a minority population of ≥30%	400,400 (67)
2 Small counties (population ≤46,500)	218,025 (37)
2 Medium-sized counties (population 46,501–130,000)	218,025 (37)
2 Large counties (population ≥130,001)	218,025 (37)
All 7 criteria combined	15,177 (3)

**Table 3:** Characteristics of Health Departments by Eligibility, Interest, and Selection Status for Randomized Trial

Characteristic	Eligible (n = 43)			Not eligible (n = 25)	No response (n = 13)
	Interested (n = 30)		Not interested (n = 13)		
	All (n = 30)	Selected (n = 6)			
Mean distance from Chapel Hill, miles	131	103	101	91	132
Mean county population	123,021	113,594	89,790	85,824	77,047
Mean % minority^a^	32	36	27	30	21
Mean per capita income, $, 2004^a^	25,724	25,823	25,786	26,475	24,935
Mean % below poverty guideline^a^	15	14	14	15	15
Mean % enrolled in Medicaid^a^	22	22	22	21	20
No. (%) participating in BCCCP^b^	27 (90)	6 (100)	12 (92)	21 (84)	8 (62)
No. (%) participating in WISEWOMAN^b^	12 (40)	2 (33)	6 (46)	9 (36)	4 (31)
Mean no. of staff^c^	138	122	108	115	83
**Mean no. (%) with full-time staff positions^d^**					
Registered dietitian	20 (67)	2 (33)	8 (62)	11 (44)	NA
Health educator	26 (87)	5 (83)	11 (85)	15 (60)	
Registered nurse assigned to patient education	21 (70)	4 (66)	10 (77)	12 (48)	
Registered nurse without health educator or registered dietitian	2 (7)	1 (17)	1 (8)	0	

Abbreviations: BCCCP, Breast and Cervical Cancer Control Program; WISEWOMAN, Well-Integrated Screening and Evaluation for Women Across the Nation; NA, not available.

a Source: North Carolina Center for Health Statistics, 2006: http://www.schs.state.nc.us/SCHS/data/pocketguide/2005/.

b Source: Personal communication with North Carolina WISEWOMAN Coordinator, North Carolina Division of Public Health, 2007.

c Source: Staffing and services fiscal year 2003 report. Division of Public Health, North Carolina Department of Health and Human Services, North Carolina Center for Health Statistics, 2004.

d Source: Health Department Capacity Survey (described in "Methods" section); 13 nonresponders.

**Table 4:** Interventionist Characteristics and Ratings of Training Topics

**Characteristic**	Mean (SD) or No. (%)
Mean (SD) years of experience working in public health	10 (9)
Mean (SD) years employed at current health department	9 (8)
No. (%) who received special training in adult weight management	1 (17)
No. (%) who developed, implemented, or evaluated a weight management intervention	4 (67)
**No. (%) with prior experience with low-income women aged 40-64 y through the following:**	
Health screening programs	4 (67)
Minority health activities	4 (67)
Women's health promotion activities	4 (67)
**Mean (SD) rating on the following training topics:^a^**	
Behavior change principles	3.5 (0.8)
Weight management counseling	3.5 (1.8)
Weight management program development	3.5 (1.8)
Community organization/mobilization	3.3 (1.0)
Body mass index measurement	2.5 (1.5)
General physical activity recommendations and guidelines	2.8 (1.0)
Weight management recommendations and guidelines	2.8 (1.5)

a Topics were ranked on a scale of 1-5; 5 indicated most important.
